# *VaSDC1* Is Involved in Modulation of Flavonoid Metabolic Pathways in Black and Red Seed Coats in Adzuki Bean (*Vigna angularis* L.)

**DOI:** 10.3389/fpls.2021.679892

**Published:** 2021-07-26

**Authors:** Liwei Chu, Pu Zhao, Kaili Wang, Bo Zhao, Yisong Li, Kai Yang, Ping Wan

**Affiliations:** ^1^Key Laboratory of New Technology in Agricultural Application, College of Plant Science and Technology, Beijing University of Agriculture, Beijing, China; ^2^State Key Laboratory of Tree Genetics and Breeding, Key Laboratory of Tree Breeding and Cultivation of the State Forestry Administration, Research Institute of Forestry, Chinese Academy of Forestry, Beijing, China; ^3^Institute of Crop Sciences, Chinese Academy of Agricultural Sciences, Beijing, China; ^4^Key Laboratory of Urban Agriculture (North) of Ministry of Agriculture, College of Bioscience and Resource Environment, Beijing University of Agriculture, Beijing, China

**Keywords:** adzuki bean, *VaSDC1*, flavonoid metabolism, seed coat colour, transcriptome, qRT-PCR

## Abstract

Seed coat colour is an important nutritional quality trait. Variations in anthocyanins and flavonoids induce the diversity of seed coat colour in adzuki bean (*Vigna angularis* L.). Red seed coat and black seed coat are important adzuki bean cultivars. Insights into the differences of flavonoid metabolic pathways between black and red adzuki bean are significant. In this study, we explored that the difference in seed coat colour between the red (Jingnong6) and the black (AG118) is caused by the accumulation of anthocyanins. The RNA-sequencing (RNA-Seq) and real-time reverse transcription (qRT)-PCR results showed that the *Vigna angularis* L. seed coat color (*VaSDC1*) gene, an R2R3-MYB transcription factor, should be the key gene to regulate the black and red seed coat colours. In three different colouring staes of seed development, *VaSDC1* was specifically expressed in the black seed coat (AG118) landrace, which activates the structural genes of flavonoid metabolic pathways. As a result, this caused a substantial accumulation of anthocyanins and created a dark blue-black colour. In the red (Jingnong6) seed coat variety, low expression levels of *VaSDC1* resulted in a lower accumulation of anthocyanins than in AG118. In addition, *VaSDC1* was genetically mapped in the interval between simple-sequence repeat (SSR) markers *Sca326-12*, *Sca326-4*, and *BAgs007* on chromosome 3 using an F_4_ segregating population derived from the cross between Jingnong6 and AG118. These results will facilitate the improvement of nutritional quality breeding in adzuki beans.

## Introduction

The adzuki bean (*Vigna angularis* L.), one of the major pulse crops in the genus *Vigna*, is mainly planted and consumed in East Asia and carries an important economic value (Yoshida et al., 2008; Shi et al., 2016). The cultivation area of adzuki bean in China is approximately 22,000 ha, making China the largest producer in the world. China is the original centre of the adzuki bean, with the largest number of genetic resources (Ning et al., 2009; Liu et al., 2013). The adzuki bean is widely used in a variety of foods (e.g., paste in pastries, desserts, cakes, porridge, adzuki bean rice, jelly, adzuki milk, ice lollies, and ice cream) for at least a billion people (Itoh et al., 2004a, b; Lestari et al., 2014). It is consumed during traditional celebrations such as the Chinese Spring Festival and Japanese weddings (Yousif et al., 2007; Horiuchi et al., 2015).

Adzuki beans are rich in starch (53.14%), protein (22.72%), iron (7.4 mg/100 g), zinc (4.0 mg/100 g), dietary fibre (12–13%), B vitamins and folic acid (Tjahjadi et al., 1988). Phenolic acids and flavonoids extracted from adzuki bean exhibited significant antioxidant, immune-regulatory and radical-scavenging activities (Amarowicz and Pegg, 2008; Yao et al., 2015). Adzuki beans possess strong 2,2′-azino-bis(3-ethylbenzothiazoline-6-sulphonic acid (ABTS^+^) free-radical-scavenging capacity and α-glucosidase inhibitory activity. Significant positive correlations (*P* < 0.01) between the antioxidant activity and total phenolic acids, and between total flavonoids and free caffeic acid contents were observed (Shi et al., 2016). The abundant phenolic substances in the adzuki bean were reported to have the strong free-radical-scavenging capacity, which can prevent and control oxidative damage caused by inflammation, atherosclerosis, cardiovascular disease and even cancer (Sato et al., 2005a, b; Hori et al., 2010).

The phenolic compounds mainly include phenolic acids and flavonoids. Flavonoids showed differences and variations during seed ripening and harvesting (Raffo et al., 2004). The phenolic content changed remarkably during the ripening process (Amiot et al., 1986; Romero et al., 2002; Bouaziz et al., 2004).

Legume seeds with dark colour (black, red and brown) possess a significantly higher phenolic acid content than lighter coloured (yellow, green and white) ones. There were numerous antioxidant activities of different legume accessions (Amarowicz and Pegg, 2008). The antioxidant activity was strongly correlated with a total phenolic acid content, and total phenolic acid content has been confirmed to correlate reasonably with seed coat colour (Xu et al., 2010). Seed coat colour influenced the synthesis and accumulation of phenolic compounds in the adzuki bean. The concentration of phenolic compounds correlated with the values of seed colour (Kim et al., 2011). Coloured seeds (e.g., black and red) had higher antioxidant activities than colourless seeds (e.g., white and beige) in common beans (Madhujith et al., 2010). We published a draft of the adzuki bean genome by whole-genome shotgun sequencing on the Jinnong6 variety (Yang et al., 2015). We analysed the genetic relationships of seed coat colour using 12 F_2_ or F_3_ hybridised combinations derived from eight seed coat colours and predicted the genetic model of adzuki bean seed coat colours, though that the difference between black and red is controlled by B locus and T/Y locus (Chu et al., 2021). The *VaScB* gene controlling the black seed coat trait (SDC1) was mapped onto chromosome 3 (Li et al., 2017). *VaScB* should be the same locus as the B locus but still needs to be verified. Black is the darkest seed coat colour in adzuki bean with the highest flavonoid content. Red is the most common seed coat colour in cultivated adzuki beans. Exploration of the regulatory mechanism between black and red seed coats and the synthesis pathway of bioactive flavonoid of adzuki bean is important to improve the antioxidant properties and quality breeding in adzuki beans.

In this study, the precise differences in gene expressions in the flavonoid metabolic pathways between red seed coat Jingnong6 and black seed coat AG118 were revealed during the different colouring stages of seed development, utilising RNA-sequencing (RNA-Seq) and real-time reverse transcription (qRT)-PCR. *VaSDC1*, an R2R3-MYB transcription factor, was suggested to lead to the difference between red and black seed coats in adzuki bean through activating the structural genes of flavonoid metabolic pathways. VaSDC1 has high homology with AtMYB75/90 in amino acid sequences that influenced the colour of Arabidopsis that was explored by phylogenetic analysis. The morphological marker SDC1 between black and red seed coats was mapped in the interval of simple-sequence repeat (SSR) markers *Sca326-12*, *Sca326-4*, and *BAgs007*, and *VaSDC1* was found in the same interval. Insight on *VaSDC1* was important to understand the regulatory mechanism of the adzuki bean seed coat colour and flavonoid metabolism. These SSR markers may be used as an assistant for the selection of the potential antioxidant properties and quality breeding in adzuki bean.

## Materials and Methods

### Plant Materials

The improved varieties of adzuki bean (Jingnong6) with red seed coat colour and the landrace (AG118) with black seed coat colour were utilised for RNA-Seq and qRT-PCR. Jingnong6, AG118 and their 209 recombinant inbred lines of F_4_ that were used for genetic mapping were grown at the Experimental Farm of Beijing University of Agriculture (BUA) in 2018. The colouration of the adzuki bean seed coat is a diffusion process from hilum to whole seed coat. We divided the colouration of adzuki bean seed coat into three stages. The hilum was only coloured in stage 1. The whole seed coat began to be coloured preliminarily in stage 2. The whole seed coat was deeply coloured in stage 3. We collected the seed coat of Jingnong6 and AG118 during the three colouring stages, respectively. All samples were immediately frozen in liquid nitrogen and then stored at −80°C.

The 209 recombinant inbred lines of F_4_ (106 dominant black seed coat individuals and 103 recessive red/light brown seed coat individuals) derived from the F_2_ population of a cross between Jingnong6 and AG118 by the single seed descent method (Horiuchi et al., 2015) were used to map the *VaSDC1* gene.

### Identification of Pigments

As described earlier (Li et al., 2020), seed coat tissues were grounded into powder; then, flavonoids and carotenoids were extracted using methanol. An equal volume of water and dichloromethane were added to the methanol extract and thoroughly mixed. Finally, the samples were centrifuged at 13,000 rpm to separate flavonoids and carotenoids into the supernatant liquid (aqueous) and the denser liquid (non-aqueous).

### Relative Quantification of Anthocyanin by Ultraviolet-Visible (UV/Vis) Spectroscopy

Seed coat tissues were grounded into powder in liquid nitrogen and then resuspended in 0.1 mol/L hydrochloric acid. The hydrochloric acid extract was sealed in a beaker, followed by a 4-h incubation at 32°C. Finally, samples were filtered and measured at a wavelength of 530 nm with an ultraviolet spectrophotometer. The absorbance of 0.1 mol/L hydrochloric acid at a wavelength of 530 nm served as the blank control. Absorbance reading of 0.1 mol/L of each sample at a wavelength of 530 nm was served as a measurement unit of anthocyanin relative content.

### Identification of Anthocyanin by Liquid Chromatography-Electrospray Ionisation–Tandem Mass Spectrometry (LC-ESI-MS/MS) Analysis

The seed coats of Jingnong6 and AG118, collected at the third colouring stages, were grounded into a powder and extracted with 1 ml of 70% aqueous methanol by overnight incubation at 4°C. The extracted solution was centrifuged for 10 min at 10,000 g. Extracts were absorbed, and the supernatant was filtered. Extracts were determined using LC-ESI-MS/MS analysis. Linear ion trap (LIT) and triple quadrupole (QQQ) scans were acquired on the Triple quadrupole-linear ion trap mass spectrometer (Q TRAP) LC/MS/MS system, operated in positive ion mode and controlled by the Analyst 1.6 software (ABI Sciex, United States). Instrument tuning and mass calibration were performed with 10 and 100 μmol/L polypropylene glycol solutions in QQQ and LIT modes, respectively. A specific set of multiple reaction monitoring (MRM) transitions was monitored for each period according to the metabolites eluted within that period.

### Total RNA and DNA Extraction

Total RNA was extracted from 0.1 g of each powdered tissue with EASYspin plus Total RNA Isolation Kit (Aidlab, China), according to the instructions of the manufacturer, with minor modifications. Genomic DNA was extracted from 0.1 g of a leaf taken from each F_4_ line by the improved cetyltrimethylammonium bromide (CTAB) method. The integrities of total RNA and DNA were further assessed by 1% agarose gel electrophoresis. Concentrations and purity of RNA and DNA were assayed by NanoDrop^TM^ 8000 Spectrophotometer (Thermo Fisher Scientific, United States).

### Illumina Transcriptome Library Preparation, Sequencing, and Expression Level Estimation

**FIGURE 1:**
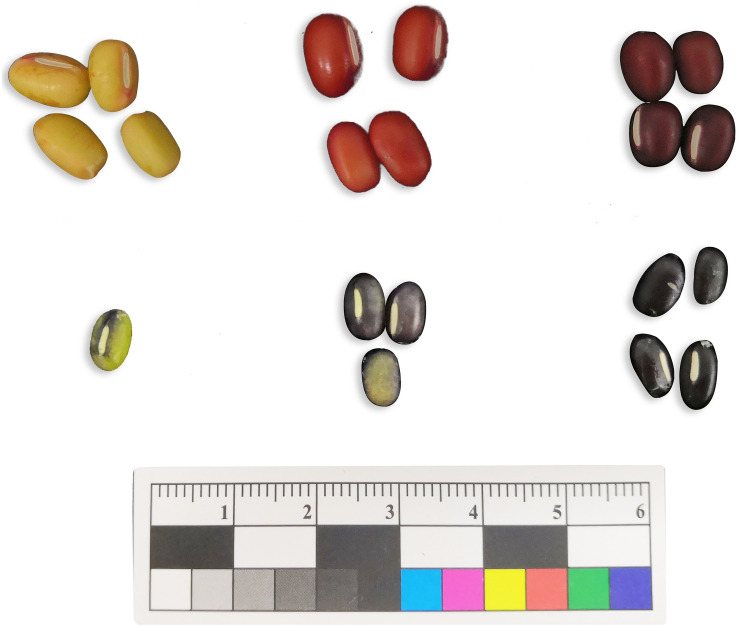
Seed coat colouring degree of Jingnong6 and AG118 in different colouring stages.

To reveal the transcriptome expression profile during seed coat colouring, total RNA from Jingnong6 and AG118 seed coats was extracted during three seed coat colouring stages and used for RNA-Seq (Figure 1). Three biological replicates were evaluated for each variety and stage, totalling 18 samples (each replicate was composed of samples mixed from more than three individuals). Messenger RNA (mRNA) was enriched by magnetic beads with oligo(dT) and fragmented with fragmentation buffer. The first-strand complementary DNA (cDNA) was synthesised by random hexamers, and the second-strand cDNA was synthesised by adding buffer, deoxynucleotide mix (dNTPs), Ribonuclease H (RNaseH) and DNA polymerase I. The purified double-stranded cDNA was then repaired at the end. A tail was added and sequenced with a sequencing connector for PCR amplification. The final products of PCR were sequenced using Illumina HiSeq^TM^ 4000. We used an in-house Perl script to process the raw data to obtain the clean data. After obtaining clean reads, hierarchical indexing for spliced alignment of transcripts (HISAT) (Kim et al., 2015) was used to compare clean reads to the reference genome (Yang et al., 2015). Gene expression levels were estimated *via* the fragments per kilobase of transcript per million mapped reads (FPKM) method using RNA-Seq by expectation-maximisation (RSEM).

### Gene Mapping

SSR Hunter version 1.3 and Oligo version 7 were used for the primer design of 12 SSR primer pairs (Supplementary Table 1). DNA was diluted to the same concentration (50 ng/μl). The reaction mixture contained 5 μl PCR mix, 1 μl DNA, 0.4 μm of each forward and reverse primer and double-distilled water (ddH_2_O) to make up the final volume to 10 μl. Amplifications were performed with the following programme: 95°C for 5 min; 40 cycles of 95°C for 30 s; 55°C for 30 s 72°C for 30 s and 72°C for 10 min. PCR products were separated on 8% polyacrylamide gel electrophoresis (PAGE), silver stained and viewed. As a morphological marker, SDC1 was used to construct a linkage map with SSR markers.

### Quantitative Real-Time PCR Analysis

The 1,000 ng of the total RNA was reverse transcribed using PrimeScript RT Reagent Kit (RR037A, TaKaRa, Japan) following the instructions of the manufacturer. The cDNA was diluted 10-fold with nuclease-free water for qRT-PCR analysis. qRT-PCR was conducted using LightCycler^®^ 96 Plates and performed on the LightCycler 96 SW 1.1 (Roche Molecular Systems, Germany). The reaction mixture contained 10 μl ChamQTM Universal SYBR^®^ qPCR Master Mix (#Q711, Vazyme, China), 1 μl 10-fold diluted cDNA, 0.4 μm of each forward and reverse primer (Supplementary Table 2) and ddH_2_O to make up the final volume to 20 μl. Amplifications were performed with the following programme: 95°C for 30 s; 40 cycles of 95°C for 10 s and 60°C for 30 s, and melting curve analysis conditions (95°C for 10 s, 65°C increased to 97°C with temperature increments of 0.11°C every 1 s). No-template reactions were used as negative controls, and each sample was assessed in three technical replicates. Gene transcription levels of seed coats from the Jingnong6 and AG118 during the three colouring stages were calculated using three biological replicates (each replicate contained the mixed seed coats of six individuals). An actin housekeeping gene of the adzuki bean was used for normalisation.

### Data Analysis

Heatmap analysis was used to display the gene expression profile using OmicShare tools.^1^ The relative expression levels of these genes were calculated according to the 2^–ΔΔCt^ method.

Neighbour-joining (NJ) phylogenetic trees were constructed with bootstrap values estimated from 1,000 replicate runs using molecular evolutionary genetics analysis version 7.0 (MEGA7) (Kumar et al., 2016) to analyse the phylogenetic relationships among the *VaSDC1* gene of the adzuki bean and the genes from *Arabidopsis.* A linkage map was constructed using JoinMap version 4.0 with a logarithm of the odds (LOD) threshold of 3.0. The Kosambi mapping function was used to convert recombination frequencies for mapping distances (Kosambi, 1944). Seed coat colour trait was calculated in the genetic map as a qualitative trait.

## Results

### Differences Between the Adzuki Bean Seed Coat Colour and Pigment Content During the Seed Colouring Stages

**FIGURE 2:**
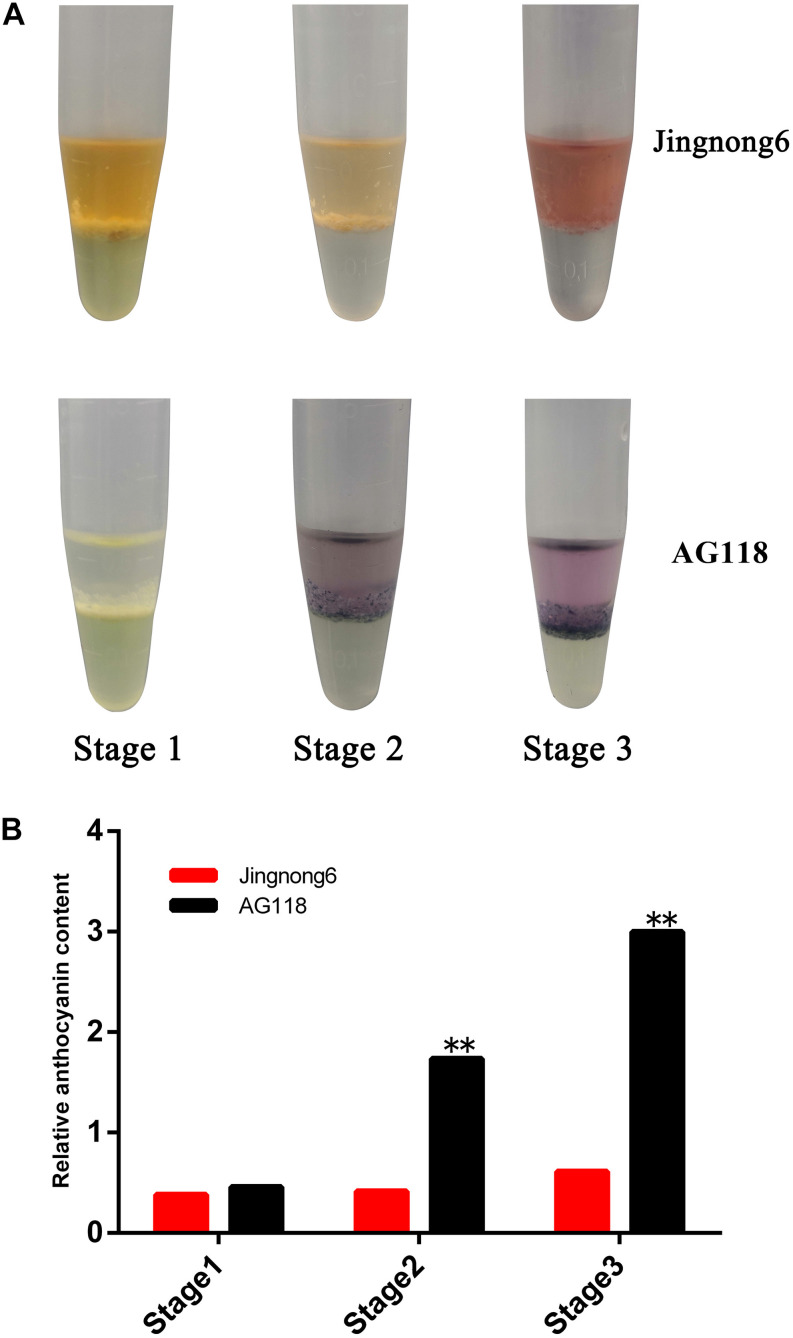
Identification of colouring substance and content in seed coats of Jingnong6 and AG118. **(A)** Identification of colouring substances in the seed coats of different colouring stages; **(B)** relative anthocyanin content of Jingnong6 and AG118 seed coats in different colouring stages. **P ≤ 0.01.

The morphological analysis showed an obvious difference in colour depth during the three seed coat colouring stages between the Jingnong6 and AG118, as the seed coat colour is gradually deepened from stage 1 to stage 3 (Figure 1). Determination of pigments showed that the difference between the red seed coat of Jingnong6 and the black seed coat of AG118 depended on anthocyanins but not carotenoids (Figure 2A).

**FIGURE 3:**
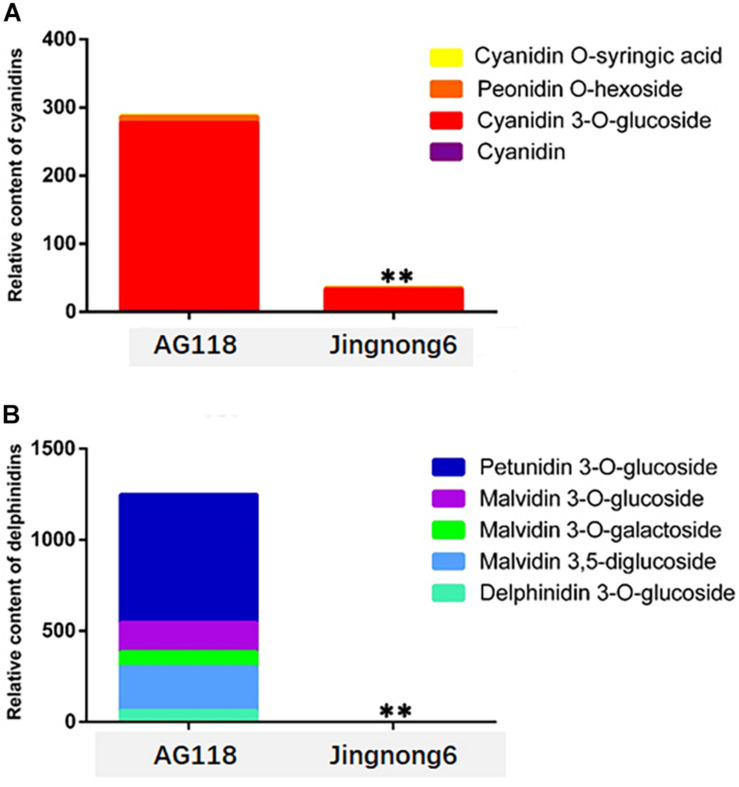
Identification and quantification of the pigments during stage 3 in seed coat colouring of Jingnong6 and AG118. **P ≤ 0.01.

In addition, the level of accumulation of anthocyanin was increased, corresponding to the colour deepening in successional colouring stages. In the AG118 landrace, the anthocyanin content quadrupled from stage 1 to stage 2 and doubled from stage 2 to stage 3. However, it only exhibited a small increase from stage 1 to stage 3 in Jingnong6 (Figure 2B). The seed coats of Jingnong6 and AG118 at stage 3 were collected and assayed for the final compositions of anthocyanins using LC-ESI-MS/MS analysis. A total of nine different anthocyanin metabolites divided into two categories were detected (Figure 3). Four kinds of centaurins were detected in stage 3 of both Jingnong6 and AG118. However, the concentration of the four centaurins in AG118 is much higher than that in Jingnong6. While five kinds of delphinidins were almost undetectable in stage 3 of Jingnong6, a much higher concentration of delphinidin was detected in stage 3 of AG118.

### Expression Profile of Structural Genes in the Flavonoid Metabolic Pathways

The RNA-Seq produced 735.1563 Mbs of clean reads from 18 libraries, and the expression level (expected number of FPKM) of each isoform was calculated with a mapping ratio of 81.24–92.56%.

**FIGURE 4:**
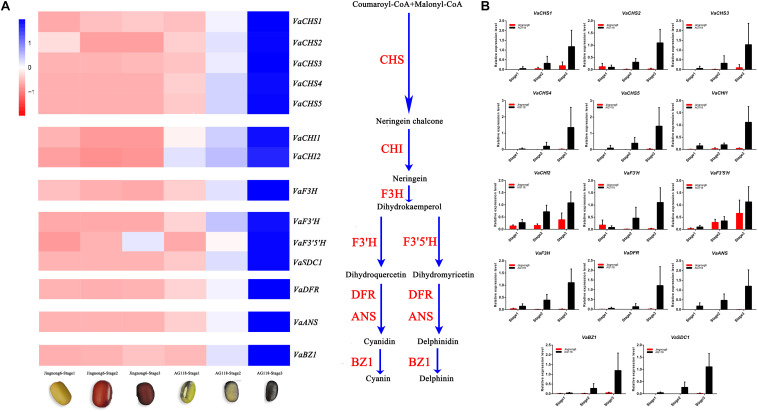
Differences in gene expression levels of Jingnong6 and AG118 seed coats. **(A)** The expression profiles of flavonoid metabolic pathway structural genes in Jingnong6 and AG118 from stage 1 to stage 3 by RNA-Seq; **(B)** the expression profiles of structural genes of flavonoid metabolic pathways verified by qRT-PCR (mean ± SD from three biological replicates).

The genes involved in the different colouring stages of Jingnong6 and AG118 were screened from the transcriptome dataset. Heatmap analysis was used to illustrate the expression profiles of Jingnong6 and AG118 structural genes in the biosynthesis of flavonoid based on their FPKM values. The results showed that multiple structural genes in the flavonoid metabolic pathways, including chalcone synthase (*VaCHS*), chalcone isomerase (*VaCHI*), flavanone 3-hydroxylase (*VaF3H*), dihydroflavonol-4-reductase (*VaDFR*), anthocyanidin synthase (*VaANS*), anthocyanidin 3-O-glucosyltransferase (*VaBZ1*), flavonoid3′-hydroxylase (*VaF3’H*), flavonoid3′5 ′-hydroxylase (*VaF3*′*5* ′*H*) and R2R3-MYB (*VaSDC1*), had different expression models in seed coat colouring stages between Jingnong6 and AG118. The expression levels of these structural genes in the flavonoid metabolic pathways observably increased from stage 1 to stage 3 in AG118 but had only small changes from stage 1 to stage 3 in Jingnong6 (Figure 4A).

To validate the RNA-Seq results, we analysed the expression levels of these structural genes from stage 1 to stage 3 in Jingnong6 and AG118 by qRT-PCR. The expression patterns of these genes were very similar to the RNA-Seq results (Figure 4B). These results validated the relevance of the RNA-Seq data. In addition, qRT-PCR results had better gene expression consistency.

### Identification of *VaSDC1* by Gene Mapping

**FIGURE 5:**
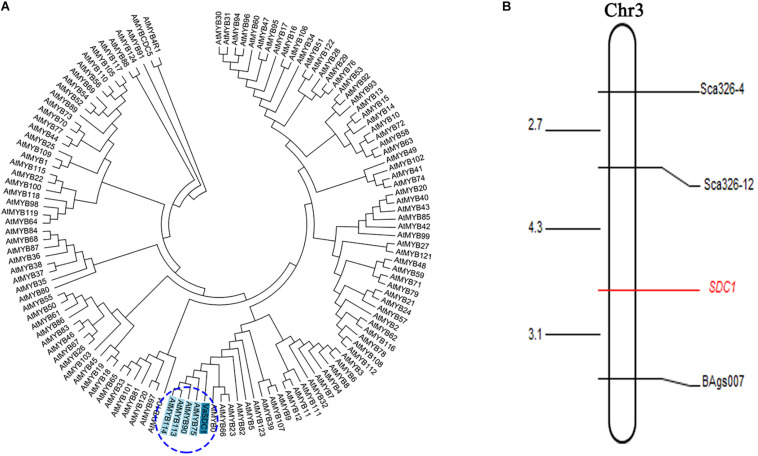
Further proof of the involvement of *VaSDC1* in the colouration difference between Jingnong6 and AG118. **(A)** Neighbour-joining (NJ) phylogenetic tree based on amino acid sequences *VaSDC1* and all R2R3-MYBs of *Arabidopsis*; **(B)** linkage map between SDC1and SSR markers.

We initially mapped the SDC1 trait (i.e., the difference between black and red seed coat colours) on a 1,454 kb physical interval between the initial position of the short arm and *s342-127390* of chromosome 3 (Li et al., 2017). *VaSDC1* was located in this range. To finely map the *VaSDC1* gene, which leads to the difference in the seed coat colour between Jingnong6 and AG118, 12 pairs of SSR primers were developed that span the 1,454 kb interval, encompassing the genomic sequence between the initial positions of chromosome 3 and *s342-127390*. Three (25%) SSR motifs were found to be polymorphic in the two parental lines (Supplementary Table 1). The colour difference between black and red seed coats was regarded as a morphological marker and named SDC1 (i.e., the difference between the black seed coat and red seed coat in F_4_ segregating population from the cross between Jingnong6 and AG118) and was used for gene mapping. Using three SSR markers, *Sca326-12*, *Sca326-4*, and *BAgs007*, the morphological marker SDC1 was found to be located between the SSR markers *Sca326-12* and *BAgs007* at a distance of 4.3–3.1 cm, respectively (Figure 5B and Supplementary Table 3).

## Discussion

The adzuki bean is also called “The Red Small Bean” in China. The seed coat colour of most cultivars is red including Jingnong6. AG118 is a rare landrace of the black seed coat. In other legume crops, seed coat colour has been shown to correlate with polyphenol content (Amarowicz and Pegg, 2008). During the colouring process of Jingnong6 and AG118, with the deepening of seed coat colour, the anthocyanin content increased gradually. The anthocyanin content of AG118 was much higher than that of Jingnong6, at each colouring stage. In stage 3, the contents of anthocyanins, delphinidins and their derivatives of AG118 seed coat were significantly higher than those of Jingnong6. Seed coat morphological analysis and anthocyanin content determination indicated that colouration of the seed coat in the adzuki bean is a process of gradual accumulation of anthocyanins.

During the three seed coat colouring stages, the expression levels of the structural genes of the flavonoid metabolic pathway in AG118 were significantly higher than that of Jingnong6 as indicated by RNA-Seq and verified by qRT-PCR. The expression levels of the structural genes in the flavonoid biosynthesis pathway can directly influence the accumulation of anthocyanin, which determines seed coat colour (Lepiniec et al., 2006). We found that expression levels of *VaF3’H* and *VaF3’5’H* were significantly higher in AG118 compared to Jingnong6 during the colouring stages. *VaF3’H* and *VaF3’5’H* are able to turn the final product into cyanidin, delphinidin and their derivatives in the flavonoid metabolic pathways but not pelargonidin. This result corresponds to our content analysis of cyanidin, delphinidin and their derivatives. Structural genes *VaCHS*, *VaCHI*, *VaF3H*, *VaDFR*, *VaANS*, *VaBZ1*, *VaF3’H*, and *VaF3’5’H* in the flavonoid biosynthesis pathway had different expression trends during the three colouring stages of Jingnong6, but their expression level increased significantly from stage 1 to stage 3 in AG118 (Figure 4). The results indicated that the expression of these genes was activated in AG118.

*VaSDC1* has the same expression pattern as these structural genes described above. *VaSDC1* is an R2R3-MYB transcription factor, which displays the highest homology with AtMYB75/90/113/114 (Figure 5A) in amino acid sequence. *AtMYB75/90* (i.e., *AtPAP1/AtPAP2*) was reported to be able to regulate the structural genes in the flavonoid metabolic pathways and further influence the colour in tomato (Li et al., 2018). In conclusion, *VaSDC1* might be the key factor that leads to the difference between black and red seed coat colours in adzuki beans. UDP-glucose:flavonoid 3-O-glucosyltransferase (*UGT78K1*) gene caused soybean black seed coat colour (Kovinich et al., 2010). *VaSDC1*, as a transcription factor, can increase the accumulation of flavonoids by activating the expression of structural genes in the flavonoid metabolic pathway and change the adzuki bean seed coat colour. *VaSDC1* is different from the *UGT78K1* gene leading to black seed coat colour in soybean. This result was significant for the breeding of antioxidant quality in adzuki beans.

Based on a previous study, we mapped the black seed coat trait SDC1, which is controlled by a single gene onto chromosome 3 (Li et al., 2017). In order to further identify whether *VaSDC1* was the key factor regulating the difference in the seed coat colour between Jingnong6 and AG118, SSR markers were used to narrow the mapping interval of SDC1 (Figure 5B). As a morphological marker, SDC1 was further mapped in the interval between *Sca326-12* (1,211,665 bp from the initial position of chromosome 3) and *BAgs007* (2,482,806 bp from the initial position of chromosome 3), which contains the *VaSDC1* gene. To a certain extent, the mapping result of SSR markers confirms our hypothesis that *VaSDC1* is the key factor regulating the colour difference between Jingnong6 and AG118. However, further verification is required.

**FIGURE 6:**
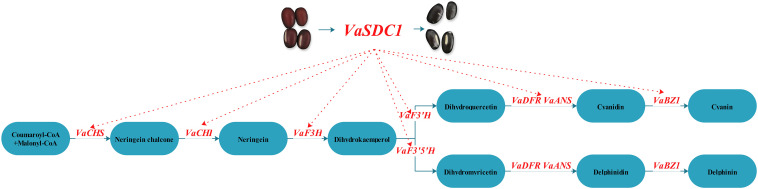
Hypothetical model of the molecular mechanism of the flavonoid metabolic pathway in the red Jingnong6 and the black AG118.

A hypothetical model of the molecular mechanism of the flavonoid metabolic pathway between black seed coat AG118 and Jingnong6 was predicted (Figure 6). The high expression of *VaSDC1* in AG118 may activate the expression of structural genes in the flavonoid metabolic pathways, further promote the accumulation of anthocyanin and lead to the difference between black AG118 and red Jingnong6. We developed three molecular markers linked to *VaSDC1.* These results enriched our understanding of seed coat colouration mechanisms. Furthermore, molecular markers can lead to the improvement of nutritional quality in adzuki beans.

## Data Availability Statement

The datasets presented in this study can be found in online repositories. The names of the repository/repositories and accession number(s) can be found below: https://bigd.big.ac.cn/gsa, PRJCA004579.

## Author Contributions

PW designed and managed the project and revised the manuscript. KY and LWC coordinated the project and experiments. LWC analysed all the data and wrote the primary manuscript. PZ collected all seed coat samples, prepared the DNA, mapped the *VSDC1* gene using SSR markers, and analysed the transcriptome. KLW took part in measuring the anthocyanin content and analysed structural gene expression levels. BZ and YSL cultivated and managed the experimental accessions, segregated population and identified the phenotypes. All authors contributed to the article and approved the submitted version.

## Conflict of Interest

The authors declare that the research was conducted in the absence of any commercial or financial relationships that could be construed as a potential conflict of interest.

## Publisher’s Note

All claims expressed in this article are solely those of the authors and do not necessarily represent those of their affiliated organizations, or those of the publisher, the editors and the reviewers. Any product that may be evaluated in this article, or claim that may be made by its manufacturer, is not guaranteed or endorsed by the publisher.

## Footnotes

1 https://www.omicshare.com/tools/home/index/index.html

## Supplementary Material

Supplementary Table 1 The description of genes and primers used in this study.

Supplementary Table 2 The description of primers for SSR markers.

Supplementary Table 3 Information about markers for gene mapping.
